# From the Inside Out: Gender Mainstreaming and Organizational Culture Within the Aid Sector

**DOI:** 10.3389/fsoc.2021.664406

**Published:** 2021-05-28

**Authors:** Michelle Lokot

**Affiliations:** London School of Hygiene and Tropical Medicine, University of London, London, United Kingdom

**Keywords:** gender mainstreaming, international non-governmental organization, organizational culture, aid–accountability, sexual exploitation and abuse, meToo

## Abstract

Many international non-government organizations (INGOs) implement interventions designed to promote gender equality, investing significant resources into embedding gender considerations into programmes through the strategy of gender mainstreaming. However, despite their altruistic mission, INGOs place less focus on addressing culture and power hierarchies within their organizations. This article suggests that many INGOs fail to walk the talk on gender equality. Through an analysis of recent challenges facing the development and humanitarian aid sector, including gaps in safeguarding and #AidToo, this paper emphasizes the importance of addressing gender equality from the inside out. It draws on feminist perspectives, the notion of the “deep structure” of organizations and the author’s own experiences to argue for the need to address gendered, racial and colonial power hierarchies within the organizational culture of INGOs. The article argues that it is no longer sufficient to reduce gender mainstreaming and inclusion to programming interventions, and that INGOs need to reflexively and intentionally tackle power and inequalities within their own culture and structures.

## Introduction

Since the Beijing Platform for Action in 1996, gender has ostensibly been “mainstreamed” within international non-government organizations (INGOs). With the increased donor focus on gender equality, INGOs have responded with countless initiatives and resources, including toolkits, checklists, guidelines and lists of gender indicators. These laudable efforts have shifted how gender-related programming is positioned within many INGOs, resulting in increased focus on the importance of designing “gender transformative” programmes (Hillenbrand et al., 2015; Brush and Miller, 2019; Kågesten and Chandra-Mouli, 2020). Visibility and resourcing have increased for issues like women’s economic empowerment, child marriage and gender-based violence. Promoting gender equality has become a requirement for many development and humanitarian programmes, requiring organizations to explain how they seek to address the complex social norms that sustain gender inequalities.

Despite some progress in positioning gender equality issues more centrally within programming, debates about the strategy of gender mainstreaming persist (Moser, 2005; Sweetman, 2012). Efforts to mainstream gender have been criticised as inconsistent and overly technocratic, influenced by results-based management approaches (Gurung et al., 2010, p. 46; Henry et al., 2017, p. 848). Scholars argue that efforts to tackle gender inequality are limited by lack of recognition of the role of formal and informal institutions in sustaining women’s subordinate position in society (Rao et al., 2016; Rao and Kelleher, 2003, p. 142). The somewhat-vague 1997 ECOSOC definition of mainstreaming as the “process of assessing the implications for women and men of any planned action” can be argued to have led to emphasis of INGO programming rather than INGO culture (UN Economic and Social Council Resolution 1997/2: Agreed Conclusions, 1997).

The cracks in the approach to gender mainstreaming are perhaps best illustrated by allegations of sexual abuse, exploitation and abuse of power within the aid sector. This viewpoint article draws on feminist perspectives, Rao and Kelleher’s 2000, Rao and Kelleher’s 2003 conceptualization of the “deep structure” of organizations, as well as the author’s personal experience as a gender specialist within INGOs. The article points to the tension between the altruistic mission of INGOs and organizational culture within INGOs. It emphasizes the need for INGOs to seek changes in norms and inequalities within their organizations, and not only in the lives of “others” who their activities aim to assist. This is not necessarily a new argument, but one warranting greater focus in the wake of aid sector scandals as well as increasing debates on decolonization and localization. This article begins by positioning #AidToo as a symptom of entrenched gender, racial and colonial inequalities and harmful social and gender norms within INGO bureaucracies. The article concludes by suggesting that INGOs would benefit from engaging in the feminist practice of reflexivity to identify how intersecting power hierarchies shape organizational culture, and should systematically assess progress in addressing power imbalances through gender and inclusion self-assessments.

### #AidToo, GENDER, RACE AND COLONIALISM

While there has been increased attention to abuse, exploitation and misconduct by aid workers in recent years, this is not a new issue. In 2002, a joint UNHCR and Save the Children United Kingdom assessment documented exploitation of refugee children by international and local NGO staff, government officials and peace-keepers in three countries in West Africa. These allegations included exchange of goods and services for sex, sounding the alarm about the need for increased protection mechanisms for communities (UNHCR and Save the Children UK, 2002). The report resulted in the creation of the Interagency Taskforce on Protection of Sexual Abuse and Exploitation (PSEA) and fed into the 2006 Core Humanitarian Standard and a slew of policies and guidelines (Ferris, 2007).

Since then, the aid sector has established processes to protect communities. However, less focus has been placed on organizational culture that may sustain such abuses. Notably, issues viewed as internal to INGOs, such as harassment of female staff by male colleagues, have not always been recognized as critical in the prevention of abuse. A 2016 survey of humanitarian aid workers found that 48% of female aid workers had experienced unwanted touching and 55% had experienced ongoing romantic or sexual advances from a male colleague (Humanitarian Women’s Network, 2016). In 2018, amidst growing global attention to abuses of power and sexual misconduct in other industries, claims arose regarding sexual exploitation by Oxfam staff in Haiti and harassment and abuse by Save the Children United Kingdom executives. As accounts of mishandling of reports and cover-ups have emerged in subsequent years, the aid sector has increased prioritization of “safeguarding” and PSEA. These efforts have resulted in organizations appointing focal safeguarding points, strengthening reporting mechanisms and whistleblowing policies, and conducting staff training. More effort has also been placed on continuing to investigate allegations, including through the UK’s International Development Committee. For example, a recent investigation implicated men from the World Health Organization and large INGOs in providing work in exchange for sex during the Ebola response (The New Humanitarian, 2020).

These examples of abuse, exploitation and misconduct demonstrate that an organization’s culture is not divorced from the social context in which it operates (Gillespie et al., 2019, p. 1). Indeed, large bureaucracies may be “hegemonic” in structure, subject to power hierarchies that constrain and shape the experiences of individuals within these structures (Gurung et al., 2010, p. 51). Since the 1970’s, feminists have pointed to the role of organizational culture, values and structures in reinforcing “masculine principles” and subordinating women (Kanter, 1977, p. 46). Such analysis has laid the groundwork for issues like sexual harassment being positioned as a consequence of organizational structure rather than the isolated acts of individuals (MacKinnon, 1979). Organizations themselves have been identified as far from gender neutral, but as structures which marginalize women and maintain gender segregation (Acker, 1990). The nature of these structures have been a subject of interest to feminists. For example, Goetz’s (1997) work on the “gendered archaeology” of organizations emphasizes that gender inequalities within organizations are not merely a result of discrimination by individuals or inadvertent policy gaps, but are embedded within structures, norms, accountability mechanisms and incentives.

Power hierarchies within organizations are not solely gendered; an intersectional approach recognizes that gender may intersect with other kinds of power hierarchies, including race (Crenshaw, 1991). While intersectionality has most often been applied to people rather than structures, there are growing calls for intersectional analysis on the racialized structures of organizations (Miller, 2020; Tariq and Syed, 2017). Within the aid sector, debates on race and humanitarianism have historically been absent (Crewe and Fernando, 2006; Kothari, 2006) though this has shifted in recent years with the calls for localization and decolonization of aid (Patel, 2020). This includes problematizing the practice of bringing international “experts” in to build capacity or lead projects, instead of relying on local knowledge (Kothari, 2005; Eade, 2007), which compounds racialized power hierarchies already present in the aid sector. Even INGO “safeguarding” efforts have been critiqued as reinforcing North-South knowledge production hierarchies and framing safeguarding without consideration of “local” knowledge (Daoust and Dyvik, 2020). Representations of “vulnerable” communities have also been criticized for perpetuating gendered and racial power hierarchies (Dogra, 2011), echoing older condemnations of the development sector (Spivak, 1994). While INGOs are beginning to reflect on the racial and colonial hierarchies they perpetuate, considering these issues from an intersectional perspective is less common compared to the efforts made solely under the banner of gender mainstreaming.

The landmark work of Rao and colleagues (2016; 2000, 2003) offers a useful framework for thinking about gender, race and colonial power hierarchies within the aid sector. They refer to the “deep structure” of organizations as the “values, history, culture and practices that form the unquestioned, “reasonable” way of work in organizations” (Rao and Kelleher, 2003, p. 143). The deep structure of organizations may be shaped by hidden and unspoken norms (Rao and Kelleher, 2000, p. 74). Deep structure may include fixed beliefs about power hierarchies, technocratic approaches to managing activities, valuing individuals over the collective (less visible) work, and failing to allow individuals to balance work and family life (Rao and Kelleher, 2000, p. 78). Their work draws attention to how a feminist commitment to devolving power and committing to participatory decision-making may clash with the focus on professionalizing the aid sector, making organizations more efficient, and creating financial accountability mechanisms to promote transparency (Rao and Kelleher, 2000, pp. 75–76).

Indeed, the focus on management by results within the INGO sector has not only directly clashed with feminist proposals to transform bureaucracies, but may also explain the failure of gender mainstreaming to adequately tackle organizational culture issues. Organizational culture may result in gender pay gaps, top-down decision-making processes, acceptability of harassment and resistance to providing parental leave beyond statutory requirements—all issues the author has witnessed within INGOs. Organizational culture can reinforce women’s subordination, mirroring the culture “outside” the organization. Despite the efforts of some development scholars and practitioners to emphasize the importance of tackling organizational culture (Sandler and Rao, 2012; Rao et al., 2016), these topics continue to slip from priority in the fixation on what is much more visible: programming. Increasingly under pressure to demonstrate “impact”, INGOs have understandably invested in ensuring programming is evidence-based. This means conducting gender assessments, developing tools for monitoring activities and evaluating interventions. Among practitioners working on gender equality issues, the need to advocate for funding and visibility also dominates. Amidst these efforts, it is perhaps inevitable that the (less visible) space of organizational culture has been de-prioritized.

### THE WAY FORWARD: TACKLING ORGANIZATIONAL CULTURE

This paper reaffirms the call for INGOs to “set their own houses in order” (Sweetman, 1997, p. 2). In the wake of increasing allegations of abuse, exploitation and misconduct within a sector that prides itself on supporting those who are less fortunate, there is a need to revisit what gender mainstreaming involves. This requires reflection on what gender mainstreaming looks like–beyond programming alone.

Expanding the interpretation of gender mainstreaming also addresses the uncomfortable hypocrisy that INGOs require gender equality to be embedded into programmes directed at distant “others”, yet may neglect inequalities within their own organizations. Staff working in INGOs are expected to implement “transformative”, participatory programming that addresses the root causes of gender inequalities. Yet, within these same organizations, staff may be subject to sexual harassment or feel they cannot negotiate for increased pay to match their colleagues. How can staff implement programmes advocating for women to have voice in decision-making in their families and communities, if they themselves do not experience inclusive decision-making in their own organizations? How can staff advocate for women and men to share caregiving and decision-making within programmes they implement, when INGOs are structured in hierarchical ways and do not intentionally seek to address the barriers preventing women from taking higher leadership positions within INGOs? These questions raise critical questions about the implications of promoting gender equality “outside” while patriarchal processes and policies rule “inside”. There exists a cognitive gap between the personal and what happens in the field (Cornwall and Rivas, 2015, p. 410) that is yet to be addressed by the strategy of gender mainstreaming.

While efforts like establishing reporting mechanisms and conducting safeguarding and PSEA training are important, these initiatives may only be a band-aid solution. Indeed, these efforts may mask underlying power hierarchies, such as those that prevent lower-level staff from reporting abuses by senior staff. Creating a reporting mechanism or increasing knowledge about how to protect individuals may not necessarily address the root causes of exploitation and abuse. Such efforts may represent a preference for a quick fix instead of sustained effort towards addressing structural issues within INGOs. The notion that establishing a safeguarding focal point or rolling out organizational training is sufficient to address the drivers of exploitation and abuse, reveals shaky underpinnings to understandings of how norms shift. Yet, in the author’s experience, training in particular continues to be emphasized as the solution to unravelling harmful organisational cultural practices and processes. Programming within INGOs has somewhat transitioned from the idea that training alone is enough to shift social and gender norms, yet even within the field of gender programming there is sometimes a preoccupation with the “individual” level of change and a neglect of structural obstacles. For example, feminist critiques highlight the problems with positioning the adolescent girl as the solution to poverty. In development and humanitarian narratives, educating girls is the silver bullet, shifting the focus from structural constraints to gender equality and power hierarchies that structure society (Hickel, 2014; Calkin, 2015; Chant, 2016; Bessa, 2019). The tendency to construct simple solutions to resolve complex problems is a common critique of the aid sector that also applies to how issues of exploitation and abuse have been addressed. While policies, reporting mechanisms and even training have their place, tackling the behaviors and norms that are grounded in gendered, racial and colonial power hierarchies inherent within the aid project, requires more intentional, longer-term work.

What might it look like to expand gender mainstreaming to issues of organizational culture? Within the literature on gender mainstreaming within organizational culture, an approach used by some INGOs and United Nations agencies is the “gender audit” or “gender self-assessment”, which uses InterAction’s methodology (Harvey, 2010). This consists of a staff survey followed by focus group discussions to benchmark efforts at gender mainstreaming. Agencies such as DFID, Oxfam and the World Bank have conducted gender self-assessments (Moser, 2005), at times adapting the methodology to include interviews with staff, a gender pay gap analysis and document review. Importantly, gender self-assessments extend beyond programming alone towards issues like perceptions of pay equity, workplace harassment, and perceptions of organizational reporting mechanisms. The process incorporates action planning, to ensure accountability to the recommendations, however these audits “are few and far between” and tend to be “once-off” (Rao and Kundu, 2016, p. 141). Nowadays, the gender self-assessment process often incorporates a focus on “inclusion”, a term which in the author’s experience is sometimes problematically conflated with intersectionality. Nevertheless, when framed with power at the centre, these self-assessments represent an opportunity for introspection (or “reflexivity” as feminists refer to it), creating the time and space for the less visible, but vitally important work of understanding power hierarchies.

## Conclusion

This paper suggests that a systematic approach to understanding power and inequalities within structures of INGOs is a constructive way to move forward with the gender equality agenda. Understanding power and inequalities intersectionally requires INGOs recognizing that they too are subject to the structures and systems of the contexts they are situated within. This requires reflection on the gendered, racialized and colonial hierarchies that may shape INGOs. Like the communities they serve, INGOs also need to engage in the sometimes-uncomfortable process of introspection. Gender and inclusion self-assessments can be an important strategy to assess progress on key areas such as technical skills, accountability, leadership and staff perceptions.

The assumption that the mission and values of INGOs to do good automatically filters into how organizations operate is one that needs to be challenged and unravelled. Organizational culture must also go beyond short-term, simplistic solutions such as one-off trainings towards more deeply reflecting on how norm change might occur at the organizational level. Here, INGOs can draw on their experiences in bringing about behavior and norm change within communities—this time applying the lessons internally instead of only through programming. Using existing group-based behavior and norm change models with INGO staff might be a useful way of starting conversations about power and creating opportunities for change.

Importantly, the process of understanding power and inequalities and addressing these issues requires a holistic consideration of organizational culture. While it may be tempting to focus more on issues like exploitation and abuse which have more visibility, INGOs must seek change across the organization. This may mean challenging perceptions that certain roles in the organization are less appropriate for female staff. It might mean INGOs being more critical of how they market themselves and depict the communities they serve. It might mean changing the approach to how senior staff are recruited and who is viewed as capable of taking on those roles. It may require changes in how INGOs fund and prioritize parental leave or mentorship programmes. More research is needed to document strategies INGOs are using to tackle power hierarchies, and to understand the mismatch between the altruistic mission of INGOs and their internal culture and power dynamics.

This article argues that narrowly consigning gender mainstreaming to programming, has resulted in a lopsided approach to understanding and unravelling complex gendered power hierarchies and norms within INGOs. Further, focusing on gender alone without recognition of how intersecting power hierarchies operate within organizations may not address the entrenched “deep structure” of organizations. Rao and Kundu’s (2016) provocative question: “What do we see when we hold the mirror up to ourselves?” may be an important starting point for INGOs (p. 136). Much more work is needed “inside” INGOs before they can hope to achieve more “outside”.

## Data Availability

The original contributions presented in the study are included in the article/Supplementary Material, further inquiries can be directed to the corresponding author.
